# Chrysomycin A Reshapes Metabolism and Increases Oxidative Stress to Hinder Glioblastoma Progression

**DOI:** 10.3390/md22090391

**Published:** 2024-08-29

**Authors:** Dong-Ni Liu, Wen-Fang Zhang, Wan-Di Feng, Shuang Xu, Dan-Hong Feng, Fu-Hang Song, Hua-Wei Zhang, Lian-Hua Fang, Guan-Hua Du, Yue-Hua Wang

**Affiliations:** 1 Beijiang Key Laboratory of Drug Target Identification and New Drug Screening, Institute of Materia Medica, Chinese Academy of Medical Sciences & Peking Union Medical College, Beijing 100050, China; liudongni@imm.ac.cn (D.-N.L.); zhangwenfang@imm.ac.cn (W.-F.Z.); fwandi@imm.ac.cn (W.-D.F.); fengdanhong@imm.ac.cn (D.-H.F.); fanglh@imm.ac.cn (L.-H.F.); dugh@imm.ac.cn (G.-H.D.); 2 Key Laboratory of Geriatric Nutrition and Health, Ministry of Education of China, School of Light Industry Science and Engineering, Beijing Technology and Business University, Beijing 100048, China; songfuhang@btbu.edu.cn; 3 School of Pharmaceutical Sciences, Zhejiang University of Technology, Hangzhou 310014, China; hwzhang@zjut.edu.cn

**Keywords:** chrysomycin A, glioblastoma, metabolism

## Abstract

Glioblastoma represents the predominant and a highly aggressive primary neoplasm of the central nervous system that has an abnormal metabolism. Our previous study showed that chrysomycin A (Chr-A) curbed glioblastoma progression in vitro and in vivo. However, whether Chr-A could inhibit orthotopic glioblastoma and how it reshapes metabolism are still unclear. In this study, Chr-A markedly suppressed the development of intracranial U87 gliomas. The results from airflow-assisted desorption electrospray ionization mass spectrometry imaging (AFADESI-MSI) indicated that Chr-A improved the abnormal metabolism of mice with glioblastoma. Key enzymes including glutaminase (GLS), glutamate dehydrogenases 1 (GDH1), hexokinase 2 (HK2) and glucose-6-phosphate dehydrogenase (G6PD) were regulated by Chr-A. Chr-A further altered the level of nicotinamide adenine dinucleotide phosphate (NADPH), thus causing oxidative stress with the downregulation of Nrf-2 to inhibit glioblastoma. Our study offers a novel perspective for comprehending the anti-glioma mechanism of Chr-A, highlighting its potential as a promising chemotherapeutic agent for glioblastoma.

## 1. Introduction

Glioblastoma (GBM) accounts for approximately 49% of malignant brain tumors, representing the predominant and a highly aggressive neoplasm of the central nervous system [1]. Surgery for gross total resection, concomitant chemoradiotherapy with temozolomide and subsequent maintenance temozolomide chemotherapy are the prevailing gold standard of treatment. Despite the available treatments, patients with GBM often have a poor prognosis with a median survival of only 14–24 months and a 5-year survival rate of about 10% [2,3]. Therefore, further research into the pathogenesis and development of novel chemotherapeutic agents for glioblastoma is a current priority.

Tumor metabolism is increasingly being recognized as a promising focus for cancer treatment strategies, which is particularly promising for the treatment of glioblastoma. The metabolic reprogramming of cancer facilitates the generation of energy (ATP) and precursors for the biosynthesis of biomacromolecules (carbohydrates, lipids, proteins and nucleic acids), supporting the rapid proliferation and metastasis of cancer cells [4,5]. The Warburg effect, a prevalent characteristic of cancer metabolism, is also called aerobic glycolysis. This alteration in metabolism is distinguished by an elevated uptake and utilization of glucose, leading to increased production of lactic acid [6]. Consequently, this leads to modifications of the tumor microenvironment through acidification and facilitates the proliferation and metastasis of cancer cells [7]. Glycolysis supplies glucose 6-phosphate to the pentose phosphate pathway (PPP), promoting nucleotide synthesis, generating reducing equivalents such as nicotinamide adenine dinucleotide phosphate (NADPH) and maintaining redox equilibrium, and supporting the biosynthesis of lipids [8]. Moreover, GBM cells are situated in an environment that is abundant in lipids; thus, lipid metabolism, including fatty acid oxidation (FAO), lipogenesis and cholesterol synthesis [9], plays a crucial role in GBM development. Among these processes, suppressing FAO is a promising cancer treatment strategy, as FAO promotes cancer growth by producing ATP and NADPH [5]. Furthermore, glutamine metabolism in tumors is increasingly recognized; the glutamine can directly participate the proline and glutathione biosynthesis pathways or undergo deamination to form α-ketoglutaric acid (α-KG), which enters into the TCA cycle to supply energy for biosynthesis [10]. With the deepening of our understanding of tumor reprogramming, interventions targeting the metabolism of specific molecules or key metabolic enzymes in the metabolic process has become an important means to treat cancer, bringing hope to patients [11]. Hence, it is crucial to investigate the aberrant metabolic mechanisms of glioma and actively search for chemotherapy drugs that can restore the normal metabolism of glioma cells.

Pharmacometabolomics, the study of the overall metabolic changes under different drug administration conditions, helps to define the metabolic signatures of drug exposure and predicting the in-depth mechanism of drug effects and drug responses [12]. Airflow-assisted desorption electrospray ionization (AFADESI)-MSI under ambient conditions is a novel technique which can realize high-coverage ambient molecular imaging with spatial information [13,14]. There are various studies that showed that this technique can image functional metabolites including neurotransmitters, organic acids, polyamines, cholines and carbohydrates in specific tissues with different diseases, such as esophageal squamous cell carcinoma [15], thyroid tumors [16] and lung cancer [17]. AFADESI-MSI could therefore help us better understand glioma’s complex biological processes and can contribute to pharmaceutical development.

Oxidative stress exerts a pivotal influence on a broad spectrum of cancers, regulating the growth, proliferation, invasion, angiogenesis and metastasis of tumor cells through abnormal alterations of signal transduction pathways. The occurrence of oxidative stress can induce apoptosis and ferroptosis, thereby diminishing the likelihood of cellular transformation and consequently impeding tumor progression [18,19]. Metabolic dysregulation is a hallmark of cancer; thus, oxidative stress is intricately linked to metabolic processes. For instance, the targeting of diacylglyceryl acyltransferase 1 disrupts lipid homeostasis, resulting in an excessive influx of fatty acids into the mitochondria for oxidation and leading to elevated levels of reactive oxygen species (ROS) production, mitochondrial impairment, cytochrome C release and apoptosis in glioma cells [20]. It is imperative to understand the interplay between oxidative stress and metabolism.

In recent decades, with the substantial rise in the abundance and variety of marine resources exhibiting various pharmacological activities, the search for anti-glioma compounds from the marine drugs is promising [21]. Chrysomycin A (Chr-A) was first discovered from Streptomyces A-419 in 1955, and is a yellow and crystalline antibiotic substance containing only carbon, hydrogen and oxygen [22]. The determination of the structure of Chr-A did not occur until 1982, which suggested that Chr-A is a benzonaphthopyranone glycoside with antibiotic activity [23]. In recent years, Chr-A was isolated from *Streptomyces* sp. 891 marine sediment [24] and was found to target glucosamine-1-phosphate acetyltransferase and tetrahydrodipicolinate N-acetyltransferase activities to inhibit peptidoglycan precursor biosynthesis, thus killing *Staphylococcus aureus*. Studies have shown that Chr-A has the potential to kill various tumors including lymphatic leukemia [25], melanoma [26] and KRAS-mutant lung adenocarcinoma [27]. Our previous studies demonstrated that Chr-A regulates the Akt/GSK-3β signaling pathway to suppress the proliferation and metastasis of U87 and U251 human glioblastoma cells and induces mitochondria-dependent apoptosis in vivo and in vitro [28,29]. Nevertheless, the potential of Chr-A to impede the progression of gliomas in an intracranial model and its specific mechanisms remain unknown.

In this study, an intracranial model established using U87 cells was used to investigate the anti-GBM effects of Chr-A. Chr-A was found to significantly suppress the GBM tumorigenicity and ameliorate the abnormal glucose, glutamate and lipid metabolism and TCA cycle activities in the glioma tissue. Four abnormally expressed metabolic enzymes, glutaminase (GLS), glutamate dehydrogenase 1 (GDH1), hexokinase 2 (HK2) and glucose-6-phosphate dehydrogenase (G6PD), which directly affect metabolites in certain pathways, were found to be downregulated by Chr-A in glioblastoma cells. Further, the levels of NADPH, which is involved in glucose and glutamate metabolism and the TCA cycle, were significantly reduced by Chr-A. Our results also showed that Chr-A could increase the accumulation of ROS and intracellular H_2_O_2_ and downregulate the expression of antioxidant proteins including Nrf-2, HO-1 and NQO-1 to trigger oxidative stress and thus curb glioblastoma progression.

## 2. Results

### 2.1. Chr-A Suppressed Orthotopic GBM Tumor Growth In Vivo

**Figure 1:**
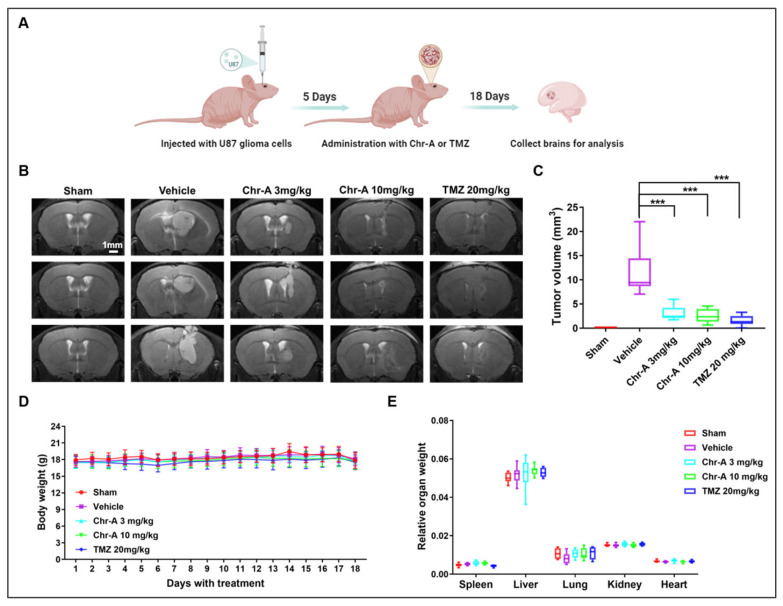
Intracranial glioma tumors. (**A**) Schematic representation of the study design to investigate the anti-GBM effects of Chr-A in an intracranial tumor model. (**B**) Representative MRI images of glioma tumors from various groups; scale bar = 1 mm. (**C**) Tumor volumes of various groups in the intracranial glioblastoma model. (**D**) Changes in body weight during the Chr-A administration. (**E**) Relative organ/weight ratio at the end of the Chr-A administration. All the data are presented as means ± SD from eight independent experiments. *** *p* < 0.001 vs. vehicle group.

To investigate the anti-GBM activity of Chr-A on glioma in an intracranial model, an orthotopic xenograft GBM model was established by implanting U87 cells into the right hemispheres of nude mice and MRI was used to monitor the tumor growth (Figure 1A). Confirming the results of our previous study [28], the Chr-A, at doses of 3 mg/kg and 10 mg/kg, caused significant glioma regression in the mice with intracranial tumors (Figure 1B,C). No significant weight loss or aberrant behavior was observed during the 18-day administration of Chr-A (Figure 1D). The organ/weight ratio in the Chr-A group also showed no significant change compared with the vehicle group (Figure 1E). These findings indicate that Chr-A exerts an inhibitory effect on glioma tumorigenesis in vivo.

### 2.2. AFADESI-MSI Revealed That Chr-A Reshapes Various Metabolism Preventing GBM Progression

**Figure 2:**
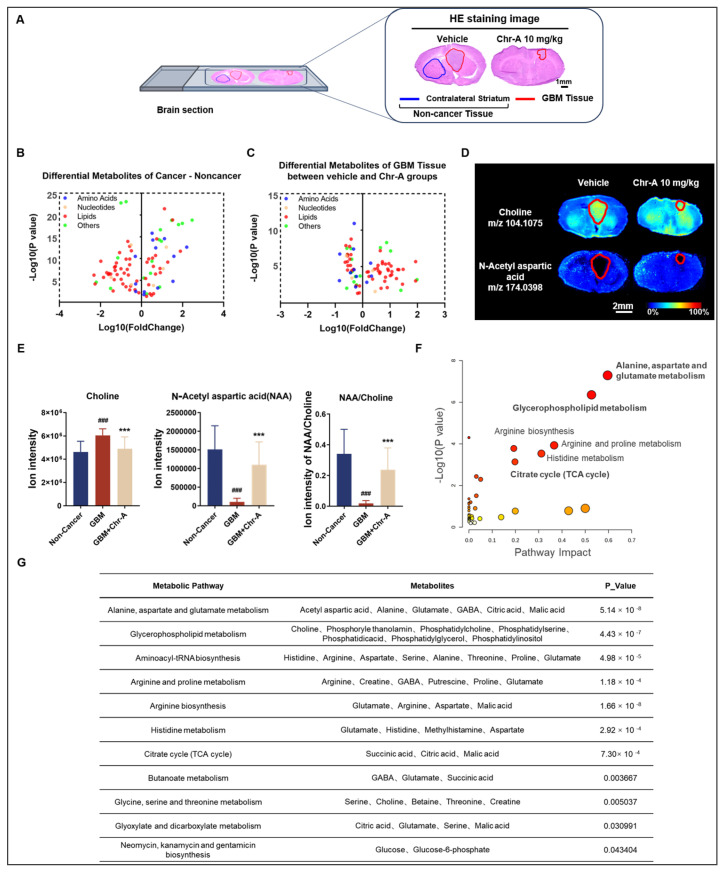
Chr-A-regulated metabolic pathways in glioma from AFADESI-MSI and MetaboAnalyst results. (**A**) Representative HE staining images. Scale bar = 1 mm. (**B**) Differential metabolites between orthotopic GBM tissues in vehicle group and non-cancerous tissues. (**C**) Differential metabolites between orthotopic GBM tissues in Chr-A group and vehicle group. (**D**) Region-specific MS images of choline and N-acetyl aspartic acid. The areas outlined in red line represent distinct tumor tissues. (**E**) Choline level, N-acetyl aspartic acid level and their ratio in non-cancerous tissues and GBM tissues with or without Chr-A. (**F**,**G**) Metabolic pathway enrichment analysis results using MetaboAnalyst. The dots’ color indicates the significance of the metabolite within the dataset (yellow = low; red = high). All the data are presented as the mean ± SD from three independent experiments. *### p* < 0.001 vs. non-cancerous tissues, *** p < 0.001 vs. GBM tissues.

Airflow-assisted desorption electrospray ionization (AFADESI)-MSI is an advanced imaging technique capable of mapping diverse functional metabolites in situ [15]. Here, in our study, the brain sections were classified into two histologic types via HE staining: non-cancerous tissues (contralateral striatum in vehicle group) and GBM tissues (tumors in vehicle group and Chr-A group) (Figure 2A).

A further analysis found that there were 97 differential metabolites between the GBM tissues of the vehicle group and the non-cancerous tissues (Figure 2B). Among these metabolites, Chr-A rescued the levels of 81 metabolites in the GBM tissues (Figure 2C). Moreover, the levels of L-carnitine C16:1, phosphatidylserine 38:2 (PS 38:2), phosphatidylethanolamine 38:4 (PE 38:4) and lysophosphatidylcholine 16:0 (lyso PC 16:0) in the GBM tissues showed significant changes with the Chr-A treatment, while four metabolites showed no difference between the GBM and non-cancerous tissues in the vehicle group (Supplementary Figure S1). L-carnitine is required for the transport of FA chains into the mitochondrial matrix, which subsequently undergo β-oxidation, thus allowing energy release from the FAs [30]. It was reported that L-carnitine could reduce apoptosis induction by carmustine in LN18 GBM cells [31]. Phosphatidylserine (PS) constitutes approximately 2–10% of the total cellular lipids [32]. In normal cells, PS is exclusively located in the inner leaflet of the cell membrane; however, in tumor cells, there is a relatively high presence of PS on the cell surface and within tumor blood vessels [33]. PS is a negatively charged phospholipid on cell membranes which is directly related to the necrosis of glioma cells caused by the membranolytic potency of mastoparan peptides [34]. PE, the second most abundant phospholipid in mammalian cells, is a potential target for glioma therapy, since it can initiate autophagy by binding to ATG8 [35,36]. Lyso PC serves as a precursor for membrane lipids that are associated with the remodeling of the plasma membrane in various types of cancer [37]. When the blood–brain barrier (BBB) is disrupted in glioblastoma, tumors are exposed to lyso PC derived from the plasma. Lyso PC serves as a potential substrate for the cell motility-stimulating factor autotaxin (ATX). Consequently, it is converted to lysophosphatidic acid (LPA) by the ATX expressed by glioblastomas, leading to the enhanced cell motility and high invasiveness of GBM [38].

A reduction in the NAA/choline ratio is a commonly used indicator for predicting increased malignancy in gliomas [39]. The U87 orthotopic tumor tissues in the right striatum showed significantly higher choline and lower N-acetyl-aspartate (NAA) levels than the non-cancerous tissues, while Chr-A rescued the NAA/choline ratio in the GBM tissues and curbed the progression of the glioma (Figure 2D,E). To identify the key metabolic pathways modulated by Chr-A in glioma, the 81 metabolites rescued by Chr-A were imported into MetaboAnalyst to perform an enrichment analysis. The analysis showed that alanine, aspartate and glutamate metabolism, glycerophospholipid metabolism, arginine biosynthesis, arginine and proline metabolism, and the TCA cycle were significantly modulated by Chr-A in GBM (Figure 2F). The different metabolites regulated by Chr-A and their metabolic pathways are listed in Figure 2G.

### 2.3. Chr-A Reduced the Abnormal Accumulation of Glucose and Glutamate and Activation of the TCA Cycle

**Figure 3:**
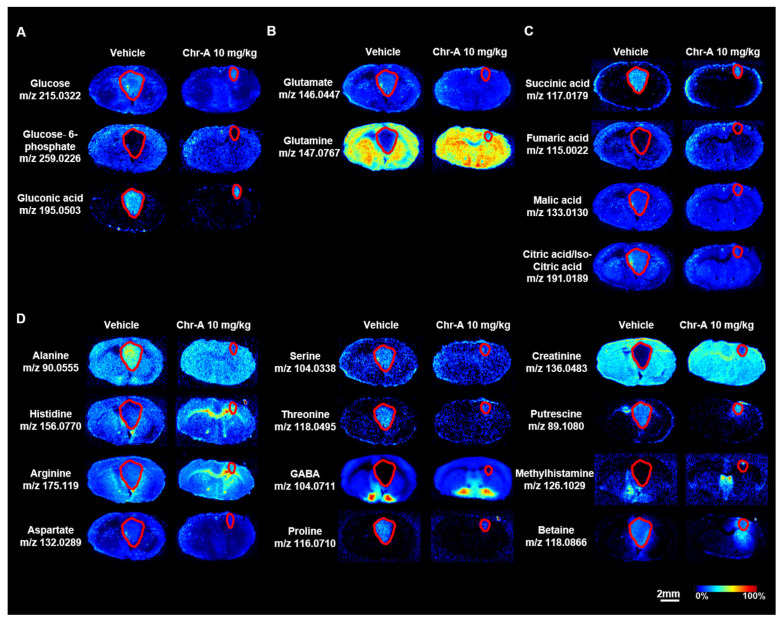
Region-specific MS images of glucose, glutamate, metabolites of the TCA cycle and other amino acids in the glioblastoma intracranial model. AFADESI-MSI showed that Chr-A reshaped the altered concentrations of glucose (**A**), glutamate (**B**), metabolites of the TCA cycle (**C**) and other amino acids (**D**) in the GBM area. The areas outlined in red represent distinct tumor tissues. Scale bar = 2 mm. All the data are from three independent experiments.

Glucose is an essential energy source for the brain to sustain neuronal activity and synaptic transmission, and the increased activity of glucose-dependent biosynthetic reactions is a hallmark of glioma tumors [4]. Glucose-6-phosphate (G6P) is the initial and pivotal metabolite of glucose metabolism, which supplies glucose to the pentose phosphate pathway (PPP) or glycolysis and the tricarboxylic acid cycle (TCA cycle), supporting the biosynthetic pathways and oxidative phosphorylation (OXPHOS) in cancers [40]. Glucose could be effectively catalyzed into gluconic acid and hydrogen peroxide (H_2_O_2_) in the presence of glucose oxidase, which regulates the tumor microenvironment (TME) by increasing the hypoxia and the acidity [41]. Using AFADESI-MSI, altered glucose, G6P and gluconic acid levels were observed in the brain tissues in situ. Compared to the non-cancerous tissues, the glucose, G6P and gluconic acid levels were increased in the GBM region (Figure 3A, Supplementary Figure S2A). With the Chr-A treatment, the abnormally increased glucose, G6P and gluconic acid levels were downregulated in the GBM region, indicating that Chr-A reduced the increase in glucose levels.

In addition to glucose, the growth of cancer cells and the maintenance of the redox state also rely on glutamine, even in glucose-deprived conditions, and glutamine starvation reduces the proliferation of glioma cells [42]. Glutamine is first converted into glutamate by glutaminase in the glutaminolysis pathway, and then enters into the TCA cycle to produce energy in the form of ATP [43]. In our study, Chr-A significantly reversed the accumulation of glutamate in the GBM, without any evident effect on the abnormally increased glutamine levels (Figure 3B, Supplementary Figure S2B).

The TCA cycle is at the energy connection point of glycolysis and glutaminolysis. Glucose enters the TCA cycle through pyruvate and glutamate enters the TCA cycle through α-ketoglutaric acid. Diverse metabolites and enzymes involved in the TCA cycle play key roles in the energy metabolism and anabolic processes of tumors [44]. Among the various metabolites in the TCA cycle, the levels of succinic acid, fumaric acid, malic acid and citric acid/isocitric acid were higher in the GBM region compared with the non-cancerous tissues, suggesting that malignant GBM is accompanied by an abnormally active TCA cycle. After treatment with Chr-A (10 mg/kg), the levels of succinic acid, malic acid and citric acid/isocitric acid were decreased compared with the vehicle group (Figure 3C, Supplementary Figure S2C). The levels of the other identified metabolites (alanine, aspartate, serine, threonine, proline, putrescine and betaine) also increased, while the levels of histidine, methylhistamine, arginine, γ-aminobutyric acid (GABA) and creatinine were reduced in the GBM tissues compared with the non-cancerous tissues. Chr-A significantly rescued the aberrant alterations of these amino acids (Figure 3D, Supplementary Figure S2D). Taken together, our results indicated that Chr-A may inhibit biosynthesis and the nutrient supply of glioma cells by disturbing glucose and glutamate metabolism and the TCA cycle, thus hindering glioma progression.

### 2.4. Chr-A Corrected Phospholipid and Fatty Acid Metabolism in Glioblastoma Xenografts

**Figure 4:**
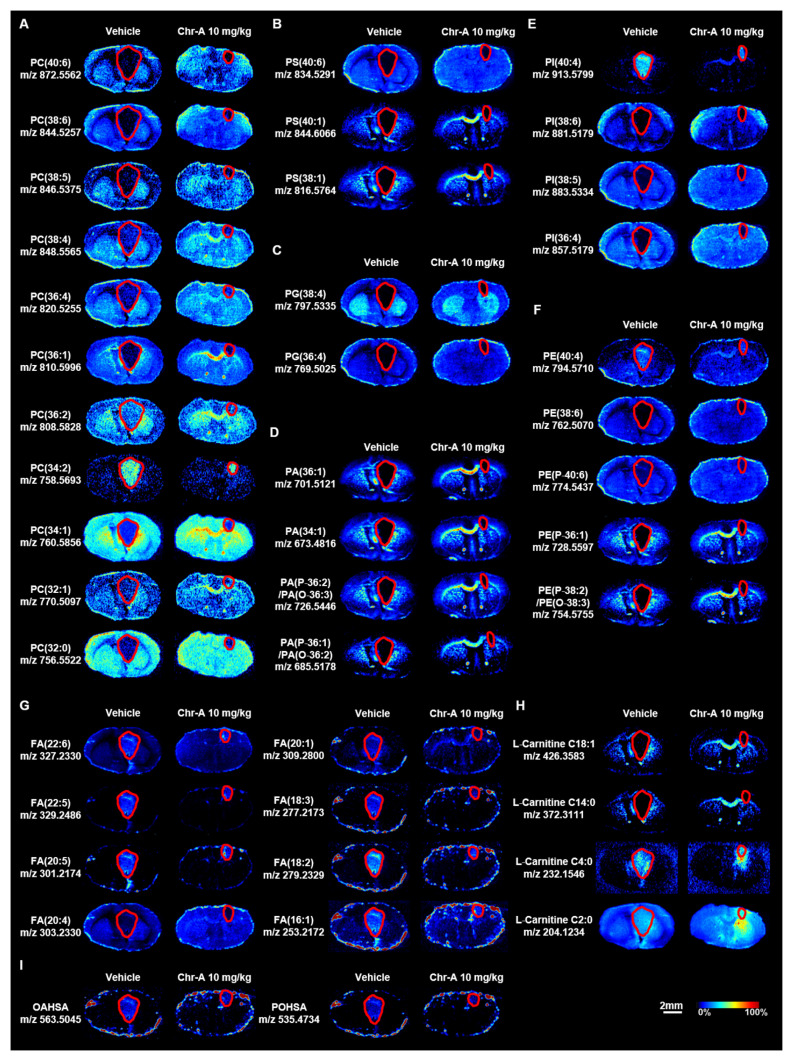
Region-specific MS images of phospholipids and fatty acids in the intracranial glioblastoma model. In situ AFADESI-MSI showed that Chr-A reversed the aberrant alteration of the contents of PCs (**A**), PSs (**B**), PGs (**C**), PAs (**D**), PIs (**E**), PEs (**F**), FAs (**G**), L-carnitine (**H**) and FAHFAs (**I**) in the GBM area. The areas outlined in red represent distinct tumor tissue. Scale bar = 2 mm. All the data are from three independent experiments.

The lipids in our body consists of fatty acids, triacylglycerides, sphingolipids, phospholipids and cholesterol. Phospholipid metabolism is significantly altered in GBM [45] and the rapid proliferation of cancer cells relies on the intricate interplay between the uptake and de novo synthesis of FAs [46], which can be exploited for therapeutic targeting of cancer. The Chr-A-regulated phospholipids in the brains with glioma were identified in situ using AFADESI-MSI. As shown in Figure 4, the levels of 11 phosphatidylcholines (PCs), 3 phosphatidylserines (PSs), 2 phosphatidylglycerols (PGs), 4 phosphatidic acids (PAs), 4 phosphatidylinositols (PIs), 5 phosphatidylethanolamines (PEs), 8 fatty acids and 4 L-carnitines were altered. Among the 11 altered PC species, the level of PC 34:2 was markedly upregulated in the glioma tissues of the vehicle group, whereas the levels of the 10 other PCs were reduced in the GBM tissues of the vehicle group compared to the non-cancerous tissues (Figure 4A, Supplementary Figure S3A). The levels of three PSs, two PGs and four PAs were dramatically reduced in the GBM tissues of the vehicle group compared to the non-cancerous region (Figure 4B–D, Supplementary Figure S3B–D). Among the four altered PI species, compared with the non-cancerous region, the level of PI 40:4 was significantly increased while those of PI 38:6, PI 38:5 and PI 36:4 was reduced in the GBM tissues (Figure 4E, Supplementary Figure S4A). The level of PE 40:4 was markedly elevated in the GBM tissues of the vehicle group compared to the non-cancerous region, whereas the levels of the four other PEs were reduced in the GBM tissues of the vehicle group (Figure 4F, Supplementary Figure S4B). The results showed that Chr-A reshaped the abnormal phospholipid metabolism in glioblastoma cells.

Moreover, the fatty acid 20:4 (FA 20:4) level was significantly decreased, and the levels of seven other FAs were increased in the GBM tissues of the vehicle group compared with the non-cancerous tissues. This showed that Chr-A markedly rescued the FA levels in the GBM tissues compared to vehicle group (Figure 4G, Supplementary Figure S4C). The carnitine system (CS) is intricately linked to the metabolic flexibility of cancer, and capable of influencing the transition between glucose and fatty acid metabolism [47]. In addition, L-carnitine is required for the transport of FA chains into the mitochondrial matrix, thus allowing energy to be released from the FAs [30]. In this study, compared to the non-cancerous tissues, the L-carnitine C2:0 and C4:0 levels were increased while the L-carnitine C18:1 and C14:0 levels were decreased in the GBM tissues and Chr-A reversed this alteration (Figure 4H, Supplementary Figure S4D).

The oleic acid ester of hydroxy stearic acid (OAHSA) and palmitoleic acid ester of hydroxy stearic acid (POHSA) are fatty acid esters of hydroxy fatty acids (FAHFAs), which exert complex effects against breast cancer and colorectal cancer development [48,49]. Using AFADESI-MSI, we found that the OAHSA and POHSA levels increased with the development of GBM, while Chr-A significantly reduced the dysregulated FAHFA levels (Figure 4I, Supplementary Figure S5). Our findings indicate that Chr-A exerts an anti-glioma effect by modulating the composition of various lipids and reshaping lipid metabolism.

### 2.5. Validation of Crucial Metabolites and Metabolic Enzymes in the Chr-A-Regulated Metabolic Pathways in Glioma In Vitro

**Figure 5:**
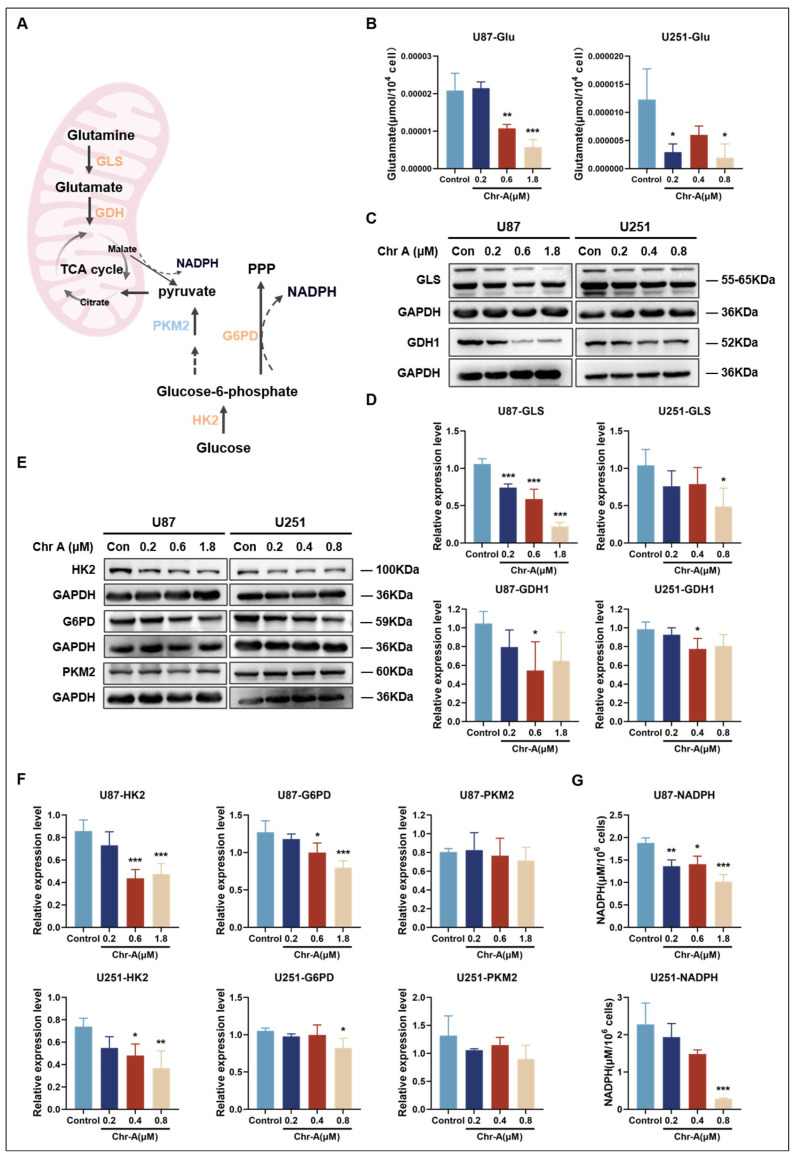
Validation of crucial metabolites and metabolic enzymes in the Chr-A-regulated metabolic pathways in vitro. (**A**) Key processes in glutamate and glucose metabolism and TCA cycle and the metabolic enzymes involved. (**B**) Glutamate (Glu) levels in U87 and U251 cells with Chr-A treatment. The data are from three independent experiments. (**C**,**D**) Chr-A significantly downregulated the protein level of the key enzymes glutaminase (GLS) and glutamate dehydrogenase (GDH1), regulating glutamine metabolism in U87 and U251 cells. The data are from four independent experiments. (**E**,**F**) Chr-A significantly downregulated the protein level of the key enzymes hexokinase 2 (HK2) and glucose-6-phosphate dehydrogenase (G6PD), but not pyruvate kinase M2 (PKM2), thereby regulating the utilization of glucose in U87 and U251 cells. The data are from four independent experiments. (**G**) Nicotinamide adenine dinucleotide phosphate (NADPH) levels in U87 and U251 cells with Chr-A treatment; the data are from three independent experiments. All the data are presented as the mean ± SD (* p < 0.05, ** p < 0.01, *** p < 0.001 vs. control group).

Glutamate is produced in mitochondria from glutamine, which is catalyzed by glutaminase (GLS), which is then converted into α-KG by glutamate dehydrogenase 1 (GDH1), fueling the TCA cycle and contributing to biosynthesis, energy generation and the cellular homeostasis of cancer cells [50] (Figure 5A). Since the glutamate levels in the GBM region were found to be significantly reduced with Chr-A treatment in vivo (Figure 3B), further detection in vitro showed that Chr-A obviously reduced the glutamate levels in U87 and U251 cells compared with the control group (Figure 5B). To better comprehend the mechanism of Chr-A-regulated glutamate metabolism in GBM, we measured the protein expression level of the key enzymes GLS and GDH1. We found that Chr-A markedly decreased the protein expression level of GLS and GDH1 in U87 and U251 cells (Figure 5C,D). Thus, Chr-A attenuated the glutamate metabolism flux in human glioma cells.

Glucose is first converted to G6P by hexokinase 2 (HK2), the most important rate-limiting enzyme of glycolysis. After that, G6P can enter the PPP through catalysis by glucose-6-phosphate dehydrogenase (G6PD) or flux into glycolysis through pyruvate kinase M2 (PKM2), another rate-limiting enzyme [51] (Figure 5A). Since the glucose and G6P levels in the GBM region were found to be reduced with Chr-A treatment in vivo (Figure 3A), the levels of HK2, G6PD and PKM2 were checked in vitro. As shown in Figure 5E,F, the expression levels of HK2 and G6PD in U87 and U251 cells were significantly decreased by Chr-A compared with the control group, while the PKM2 level showed no change. These results indicated that Chr-A could inhibit the glucose flux to the PPP. PPP signaling generates reduced equivalent nicotinamide adenine dinucleotide phosphate (NADPH) and ribose 5-phosphate, which are necessary for the synthesis of nucleotides, coenzymes and essential amino acids, especially during glucose starvation [52,53]. In our study, the level of NADPH was notably reduced due to the inhibition of the PPP by Chr-A in glioma cells (Figure 5G). Thus, Chr-A prevented the utilization of glucose by glioma cells through the inhibition of HK2 and G6PD.

### 2.6. Chr-A Increased Oxidative Stress in Glioma Cells

Oxidative stress refers to a dysregulation between reactive oxygen species (ROS) and antioxidants [54]. NADPH produced by G6PD in the PPP supports cells in responding quickly to oxidative stress through metabolic reprogramming after acute exposure to ROS [18]. Moreover, malate in the TCA cycle, derived from glutamate-converted α-ketoglutarate, can shuttle to the cytosol and produce NADPH to ameliorate an aberrant redox state [55]. As mentioned before, the TCA cycle is fueled by glutamate flux and the PPP was significantly inhibited by Chr-A. Specifically, malic acid levels, the expression level of G6PD and NADPH production were significantly reduced by Chr-A, indicating that Chr-A may mediated oxidative stress in glioma cells through metabolic reprogramming.

**Figure 6:**
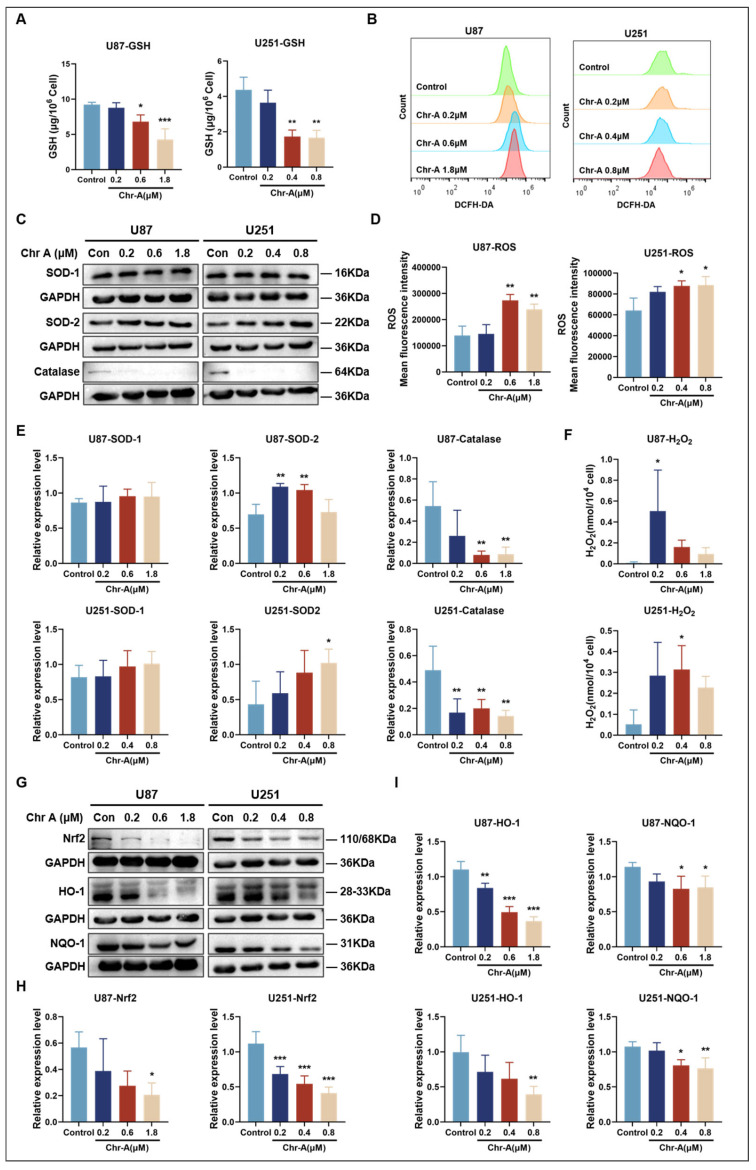
Chr-A increased oxidative stress in vitro. (**A**) Glutathione (GSH) levels in U87 and U251 cells with Chr-A treatment. The data are from three independent experiments. (**B**,**D**) Chr-A significantly increased ROS accumulation in U87 and U251 cells. The data are from three independent experiments. (**C**,**E**) Key enzymes involved in oxidative stress in U87 and U251 cells. The data are from four independent experiments. (**F**) H_2_O_2_ levels in U87 and U251 cells with Chr-A treatment. The data are from three independent experiments. (**G**–**I**) Chr-A notably downregulated the expression of Nrf-2, HO-1 and NQO-1 in U87 and U251 cells. The data are from four independent experiments. All the data are presented as the mean ± SD (* p < 0.05, ** p < 0.01, *** p < 0.001 vs. control group).

NADPH supports tumorigenesis by downregulating ROS levels, functioning as a reducing cofactor for GSH synthesis in cells [56]. In our study, the GSH levels in U87 and U251 cells were significantly decreased by Chr-A (Figure 6A), suggesting that Chr-A increased oxidative stress in GBM cells to prevent tumor progression. Further, the increased ROS levels in glioma cells due to Chr-A supports this inference (Figure 6B,D). In addition to GSH preventing oxidative damage by acting as an electron donor, enzymes such as superoxide dismutase (SOD) and catalase are involved in the removal of free radicals and reactive species, which can be triggered by ROS [57,58]. As shown in Figure 6C,E, Chr-A triggered the upregulation of SOD-2 expression and downregulation of catalase expression, while there was no effect on the expression level of SOD-1 in U87 and U251 cells compared to the control group. Superoxide anion is converted to H_2_O_2_ under the action of SOD and H_2_O_2_ can be further catalyzed to water and oxygen by catalase [59]. Therefore, we speculated that Chr-A causes the accumulation of H_2_O_2_ in GBM cells, leading to oxidative damage, which was confirmed by our results (Figure 6F).

Nuclear factor erythroid 2-related factor 2 (Nrf2), is a master regulator of redox homoeostasis [60]. After the Chr-A treatment, the expression level of Nrf2 and its downstream targets heme oxygenase 1 (HO-1) and quinone oxidoreductase 1 (NQO-1) were significantly downregulated in U87 and U251 cells (Figure 6G–I). Taken together, these results suggested that Chr-A regulates metabolic reprogramming, consuming NADPH and depleting GSH, and causing Nrf2-mediated oxidative stress in GBM cells.

## 3. Discussion

Although Chr-A has been proven to attenuate proliferation, induce DNA damage, promote apoptosis and cause cell cycle arrest, inhibiting the progression of various tumors, such as leukemia [61], KRAS-mutant lung adenocarcinoma [27] and glioblastoma [28,29], the antitumor effects of Chr-A in a glioma orthotopic model and whether Chr-A regulates metabolism reprogramming were previously unclear. In this investigation, the compound Chr-A was demonstrated to have inhibitory effects on glioma growth in intracranial xenograft models for the first time. Using AFADESI-MSI, the metabolic landscape, including altered glucose metabolism, glutamate metabolism, TCA cycle, lipid metabolism and other amino acid or small molecule metabolism, in glioma-xenograft mice treated with Chr-A was mapped. Furthermore, four abnormally expressed metabolic enzymes that directly regulate metabolites (GLS, GDH1, HK2 and G6PD) were downregulated by Chr-A in glioblastoma cells. Moreover, Chr-A reduced NADPH levels, further increased the accumulation of ROS and intracellular H_2_O_2_ and downregulated antioxidant proteins, including Nrf-2, HO-1 and NQO-1, to trigger oxidative stress and thus curb glioblastoma progression.

The metabolic heterogeneity of cancer is an important factor hindering therapeutic efficacy and its prognosis. The emergence of spatial metabolomics facilitates the understanding of the modified crosstalk among diverse metabolites in situ; these studies use advanced technology, such as matrix-assisted laser desorption ionization mass spectrometry imaging (MALDI-MSI) and AFADESI-MSI [62]. There have been several MALDI-MSI-based glioma studies in the past [63,64,65], which detected differentially distributed functional metabolites in different tumor areas within the glioma. We used AFADESI-MSI to characterize the metabolism of glioblastoma in situ, and to explore the mechanism of Chr-A remodeling of glioma metabolism for the first time. N-acetylaspartate (NAA), produced from aspartate and acetyl-coenzyme A in neurons, serves as a marker for neuronal health [66]. In accordance with prior research findings, our study observed a decrease in NAA levels in U87 orthotopic tumor tissues compared to non-cancerous tissues. Furthermore, treatment with Chr-A significantly reversed these changes in NAA levels in glioblastoma tissues, thereby impeding the progression of the glioma (Figure 2D,E).

Otto Warburg was the first to demonstrate that cancer cells increase their glucose consumption and produce lactate, even under aerobic conditions, which is a hallmark of cancer [67]. Glycolysis provides glucose 6-phosphate to the PPP for biomass synthesis, which in turn produces NADPH and precursors essential for nucleic acid biosynthesis. Moreover, cancer cells also depend on glutamine and one-carbon metabolism to sustain various metabolic functions, even under glucose-deprived conditions [40,42,50]. In agreement with the published literature [36,63], increased glucose, glucose 6-phosphate, glutamine and glutamate levels in glioma tissues were observed. In addition, an elevation in the levels of succinic acid, fumaric acid, malic acid and citric acid/isocitric acid, which are part of the TCA cycle, was also detected in glioma tissues, suggesting that the glycolysis rate exceeded the TCA cycle rate [68]. Glucose and glutamine metabolism support tumor energy generation, and serve as significant sources of nitrogen and carbon for the biosynthesis of amino acids, lipids and nucleotides [69]. Our results indicated that Chr-A reversed the increased glucose and glutamine metabolism and TCA cycle activity, hindering energy generation and biological synthesis to curb glioblastoma progression (Figure 3).

Phospholipids play a pivotal role in the formation of the lipid bilayer in biofilms, as well as in maintaining the structural integrity of cells to support rapid proliferation. Additionally, they have diverse functions including acting as second messengers, protein chaperones and receptors that are recruited by membrane-binding proteins during processes such as cell division, autophagy and apoptosis [70]. Targeting the TCA–phospholipid–glycolysis metabolism axis is effective in reshaping tumor energy metabolism and ATP production, causing G0/G1 cell cycle arrest and thus suppressing glioblastoma in vivo and in vitro [71]. Elevated PC metabolism is a common characteristic of breast, prostate and colorectal cancer and different types of brain tumors [72,73]. Interestingly, in our study, with the exception of PC 34:2, the abundance of the remaining ten PCs showed a significant decrease in gliomas (Figure 4A). Our findings are partially corroborated by a study utilizing MALDI-MSI to investigate lipid metabolism in gliomas [64]. PEs, PSs, PIs, PAs and PGs with important physiological functions also play essential roles in lipid metabolism. The dysregulation of the PC to PE ratio in the cellular membrane triggers apoptosis [74]. The application of AFADESI-MSI mapped a more comprehensive landscape of phospholipid metabolism in gliomas. In addition to the significant increase in the abundance of PE 40:4 and PI 40:4 in gliomas, there was a notable decrease in the abundance of four PEs, five PSs, three PIs, four PAs and two PGs, which was reversed by Chr-A (Figure 4B–F). In summary, Chr-A altered phospholipid levels to reshape phospholipid metabolism, rendering cancer cell membranes more susceptible to cell death and thus hindering tumorigenesis.

The enhanced flux of glucose- and glutamine-derived carbons into fatty acid synthesis resulting in almost complete dependence on de novo biosynthesis to support rapid growth and proliferation is associated with the malignancy of tumors [75]. Within cells, FAs participate in membrane formation or oxidation to carbon dioxide to provide an energy source. GBM tumors rely on fatty acid oxidation (FAO) for respiration and proliferation, and inhibiting FAO can extend the survival time of a malignant glioma mouse model [76]. Targeting multiple points within FA metabolism, including uptake, synthesis, degradation, storage and release, to subvert rapid proliferation is a promising method to treat various cancers and there have been many chemical inhibitors available for specific steps in this process [77]. The abundance of FAs (22:6, 22:5, 20:5, 20:1, 18:3, 18:2, 16:1) was found to be elevated in glioma tissues compared with the normal region, indicated that GBM holds sufficient energy resources to facilitate growth and this could be reversed by Chr-A (Figure 4G). Energy release from FAs requires L-carnitine to enter into mitochondria [30]. Correspondingly, L-carnitine (C2:0, C4:0) levels were increased in the GBM region and this was reversed by Chr-A (Figure 4H). These results imply that Chr-A reshapes fatty acid metabolism in glioblastoma cells.

Critical enzymes directly associated with the significantly dysregulated metabolites are extensively involved in tumor progression and are potential targets for diagnosis and treatment. Glutamine is converted to glutamate by GLS in mitochondria, which is further converted to α-KG by GDH1 [43]. Studies showed that inhibition of GLS and GDH1 significantly reduced the tumorigenesis of glioblastoma cells [78,79]. Under conditions of glucose deprivation, glutamine is converted to malic acid in the TCA cycle by α-KG; the malic acid is transported to the cytosol to produce NADPH, decreasing the ROS levels in cells [55]. HK2 mediates the rate-limiting step of glucose conversion to glucose 6-phosphate, which inactivates GSK-3β, promoting epithelial to mesenchymal transitions [80]. G6PD catalyzes the first step in the PPP, supporting growth. The PPP is the major pathway for generating reducing equivalent NADPH, conferring resistance to oxidative stress [81]. Dysregulated G6PD activation promotes cell proliferation in various cancers including glioma [82]. This study suggests that Chr-A markedly reduces the expression levels of GLS, GDH1, HK2 and G6PD in U87 and U251 glioma cells, which support the significant metabolic pathways identified through AFADESI-MSI, suggesting that these key enzymes might be potential targets for Chr-A (Figure 5C–F).

Our previous studies suggested that Chr-A downregulates protein kinase B (Akt)/glycogen synthase kinase 3 β (GSK-3β) and the downstream c-Myc to inhibit the growth of glioma cells in vivo [28] and in vitro [29]. AKT is a critical regulator of various metabolic enzymes and pathways and the activation of AKT drives glucose uptake, leading to the elevated glycolytic metabolism observed in various cancer cells [83], and promotes hexokinase 2 (HK2) activity, providing an abundant mitochondrial source of ATP for rapid HK2-mediated glucose phosphorylation [84]. Under aerobic glycolysis conditions, the phosphatidylinositol-3 kinase (PI3K)–AKT pathway may promote glutamine metabolism through MYC activation, thereby maintaining TCA cycle flux [85]. Moreover, AKT also regulates metabolic reprogramming by activating regulatory transcription factors or key downstream effectors that play major roles in cellular metabolic reprogramming, including mTORC1 and glycogen synthase kinase 3 (GSK3) [86]. For instance, GSK3, the first AKT substrate to be discovered, is a key regulator of cellular metabolism and was initially found to block glycogen synthesis through the phosphorylation and inhibition of glycogen synthase [87]. In addition, AKT signaling also activates the sterol regulatory element-binding protein family of transcription factors, inducing the expression of nearly all enzymes of fatty acid and sterol synthesis to promote de novo lipid synthesis [88,89]. Thus, we speculate that Chr-A may affect the glucose, glutamate and lipid metabolic pathways through AKT signaling to regulate metabolic reprogramming in glioma cells and play an anti-GBM role.

NADPH, an essential antioxidant, is partially produced through the PPP [81] and partly through the cytoplasmic shuttling of malic acid [55]. The synthesis of another antioxidant, GSH, is closely linked to NADPH and acts as a crucial reducing cofactor [56]. Both NADPH and GSH play vital roles in mitigating the accumulation of reactive oxygen species (ROS) and maintaining cellular redox homeostasis. The role of ROS in tumor growth is dual: at low or moderate levels, it can promote tumor formation and progression, whereas at high levels, it can induce cell death and severe cellular damage [90]. The decreased level of NADPH and GSH along with the accumulation of ROS in U87 and U251 cells, suggests that Chr-A significantly enhances oxidative stress in glioma cells (Figure 5G and Figure 6A,B,D). This is further supported by the upregulation of SOD-2, downregulation of catalase and accumulation of H_2_O_2_ (Figure 6C,E,F). Interestingly, it has also been confirmed that Chr-A induces the accumulation of ROS, causing DNA damage in KARS-mutated lung adenocarcinoma [27] and Staphylococcus aureus [91], suggesting that Chr-A exerts various pharmacological activities through modulating ROS levels. Therefore, we hypothesize that ROS may be a potential target of Chr-A. Nrf-2, which is activated in multiple tumors [92], is a transcription factor that can increase the generation of antioxidant proteins such as HO-1 and NQO-1 to maintain the redox balance [60]. Moreover, Nrf-2 is the sole controller of the glutamate–cysteine ligase (GCL) complex, which monitors the rate-limiting substrate of GSH synthesis [93]. Interestingly, Nrf-2 promotes the PPP and NADPH production by targeting six genes involved in the two pathways and enhances purine nucleotide synthesis and glutamine metabolism in the presence of active PI3K–Akt signaling in cancer cells [94]. Thus, Nrf-2 not only directly regulates antioxidant factors, but also impacts metabolic reprogramming including glucose and glutamine metabolism and NADPH and GSH production to maintain the redox balance in tumor cells. In our study, Chr-A significantly downregulated the expression of key proteins regulating metabolism reprogramming and oxidative stress. Nrf-2 was significantly reduced in glioma cells. This reduction also affected the expression of the downstream proteins HO-1 and NQO-1 (Figure 6G–I).

Taken together, Chr-A (3 mg/kg and 10 mg/kg) markedly inhibited the glioma growth in an orthotopic glioma xenograft model. The AFADESI-MSI results showed that Chr-A could reshape glucose and glutamate metabolism, the TCA cycle, lipid metabolism and the levels of other amino acids in gliomas. Chr-A was also shown to increase oxidative stress by regulating the expression level of Nrf-2 and decreasing NADPH and GSH levels. These findings contribute to a better understanding of the spatial metabolic characteristics of gliomas and to identifying novel metabolic targets. Additionally, this work offers new insights into the mechanism of Chr-A against gliomas, which is valuable for advancing anti-tumor studies on marine drugs.

## 4. Materials and Methods

### 4.1. Chemical Reagents

The primary antibodies used in this study can be found in Supplementary Table S1, while the other reagents used in this study can be found in Supplementary Table S2.

### 4.2. U87 and U251 Cell Culture

The source and culture conditions for the U87 and U251 cells were described previously [15].

### 4.3. U87 Glioma Orthotopic Model in Nude Mice and Drug Administration

The animal studies were approved by the Ethics Committee for Laboratory Animal Care. Female BALB/c nude mice weighing 17–19 g were purchased from Beijing Vital River Laboratory Animal Technology Co., Ltd. (Beijing, China; animal certification number SCXK (Jing) 2021-0006). U87 cells were harvested and slowly transplanted into the brain to induce tumorigenesis. Five days later, an intracranial tumor formed and the mice were randomly divided into five groups including a sham group, vehicle group, Chr-A (3 mg/kg) group, Chr-A (10 mg/kg) group and temozolomide (TMZ, 20 mg/kg) group. Chr-A and TMZ were prepared with physiological saline containing 0.1% Tween 80. Chr-A, at doses of 3 mg/kg or 10 mg/kg, was injected intraperitoneally once daily and TMZ was orally administered once per day for 18 days. At the end of the experiment, all the mice were euthanized. MRI and AFADESI-MSI were used to image the tumor in the mice and the metabolites.

### 4.4. MRI Analysis

An MRI scanner was employed for the imaging and detection of brain tumors after the administration of Chr-A for 17 days. The parameters for the MRI were as follows: T2_TurboRARE (E2), 0.5 mm slice thickness, TR/TE 2795/35, 4 averages and 22 × 22 fields of view. The slices were analyzed using RadiAnt DICOM Viewer software (version 2022.1).

### 4.5. AFADESI-MSI Analysis

The brain in the vehicle and Chr-A 10 mg/kg groups (3 mice per group) were sliced along the coronal plane into 12 μm-thick sections using a CM 1860 UV cryostat microtome (Leica, Wetzlar, Germany). The contralateral striatum area was regarded as non-cancerous tissue and the tumor region was regarded as GBM tissue in the analysis. The AFADESI-MSI analysis and data processing were performed as described previously [30]. The parameter settings are shown in Table 1 and Table 2.

### 4.6. Metabolic Pathway Enrichment Analysis

Differential metabolites were analyzed using MetaboAnalyst 5.0 (https://www.metaboanalyst.ca/, accessed on 23 August 2023) to determine the key metabolic pathways regulated by Chr-A in the GBM area.

### 4.7. Intracellular Metabolite Measurements

The intracellular levels of glutamate (Glu), glutathione (GSH) and hydrogen peroxide (H_2_O_2_) were determined using commercial kits (Solarbio, Beijing, China) following the manufacturer’s instructions. The intracellular level of nicotinamide adenine dinucleotide phosphate (NADPH) was determined using commercial kits (ApplyGene, Beijing, China) following the manufacturer’s instructions.

### 4.8. ROS Flowcytometry

A ROS Detection Kit (Beyotime Biotechnology, Shanghai, China) was used to determine the level of ROS accumulation in U87 and U251 cells after the Chr-A treatment. In brief, cells were seeded into 6-well plates, and Chr-A was added for 1 h. Then, the cells were processed according to the instruction manual.

### 4.9. Western Blotting Assay

After treatment of Chr-A for 48 h, the total proteins in the U87 and U251 cells were extracted followed by quantification with a BCA Protein Assay Kit. Subsequently, SDS-PAGE was used to separate the proteins. After incubation with primary antibodies, the membranes were processed to visualize the protein bands. The images were analyzed as described previously [15].

### 4.10. Statistical Analysis

The results are analyzed using one-way ANOVA with Dunnett’s multiple comparisons test using GraphPad Prism 9.0 and are represented as the mean ± SD.

## Figures and Tables

**Table 1:** Basic parameters of AFADESI ion source.

Parameter	Value
Spray voltage	4500 V
Tube voltage	0 V
Spray solvent flow	6 μL/min
Spray gas pressure	0.7 MPa
Extracting gas flow	25 L/min
Spray angle	60°
*X* axis move speed	0.1 mm/s
*Y* axis step size	0.1 mm
Distance from sprayer to surface	0.7 mm
Distance from sprayer to tube	3 mm
Distance from orifice to guide tube	10 mm
Spray solution	ACN/H_2_O (8:2, *v*/*v*)

**Table 2:** Basic parameters of MS.

Parameter	Value
Polarity	Positive/Negative
Scan mode	Full scan SIM
Scan range	70–1000 Da
Capillary temperature	350 °C
Sheath gas flow rate	0 L/min
Aux gas flow rate	0 L/min
Sweep gas flow rate	0 L/min
Aux gas heater temperature	0 °C
Maximum inject time	200 ms for Full MS
AGC target	3 × 10^6^
Resolution	70,000
Scan rate	~2.2 scans/s

## Data Availability

The original data presented in the study are included in the article and Supplementary Materials; further inquiries can be directed to the corresponding author.

## Supplementary Materials

The following supporting information can be downloaded at: https://www.mdpi.com/article/10.3390/md22090391/s1, Figure S1: Region-specific MS images of L-carnitine (C16:1), PS (38:2), PE (38:4) and lyso PC (16:0) levels in the glioblastoma intracranial model; Figure S2: The ion intensity of metabolites from various metabolic pathways, as determined by AFADSI-MSI; Figure S3: The ion intensity of PCs, PSs, PGs and PAs, as determined by AFADSI-MSI; Figure S4: The ion intensity of PIs, PEs, FAs and L-carnitines, as determined by AFADSI-MSI; Figure S5: The ion intensity of OAHSA and POHSA determined by AFADSI-MSI; Table S1: Information on antibodies used in the experiments; Table S2: Information on other reagents used in the experiments.
