# Supplementary material for: Chrysomycin A Reshapes Metabolism and Increases Oxidative Stress to Hinder Glioblastoma Progression
Source: Mar Drugs. 2024 Aug 29;22(9):391. doi: 10.3390/md22090391 (PMC11433325; doi:10.3390/md22090391)
Supplement: Supplementary file 1 [file marinedrugs-22-00391-s001.zip › marinedrugs-3150248-supplementary.pdf]

Supplementary Materials for

Chrysomycin A Reshapes Metabolism and Increases Oxidative Stress to Hinder Glioblastoma Progression

Dong-Ni Liu [1], Wen-Fang Zhang [1], Wan-Di Feng [1], Shuang Xu [1], Dan-Hong Feng [1], Fu-Hang Song [2], Hua-Wei Zhang [3], Lian-Hua Fang [1], Guan-Hua Du [1] and Yue-Hua Wang [1,*]

[1] Beijiang Key Laboratory of Drug Target Identification and New Drug Screening, Institute of Materia Medica, Chinese Academy of Medical Sciences & Peking Union Medical College, Beijing 100050, China; liudongni@imm.ac.cn (D.-N.L.); zhangwenfang@imm.ac.cn (W.-F.Z.); fwandi@imm.ac.cn (W.-D.F.); fengdanhong@imm.ac.cn (D.-H.F.); fanglh@imm.ac.cn (L.-H.F.); dugh@imm.ac.cn (G.-H.D.)

[2] Key Laboratory of Geriatric Nutrition and Health, Ministry of Education of China, School of Light Industry Science and Engineering, Beijing Technology and Business University, Beijing 100048, China; songfuhang@btbu.edu.cn

[3] School of Pharmaceutical Sciences, Zhejiang University of Technology, Hangzhou 310014, China; hwzhang@zjut.edu.cn

* Correspondence: authors: wangyuehua@pku.org.cn

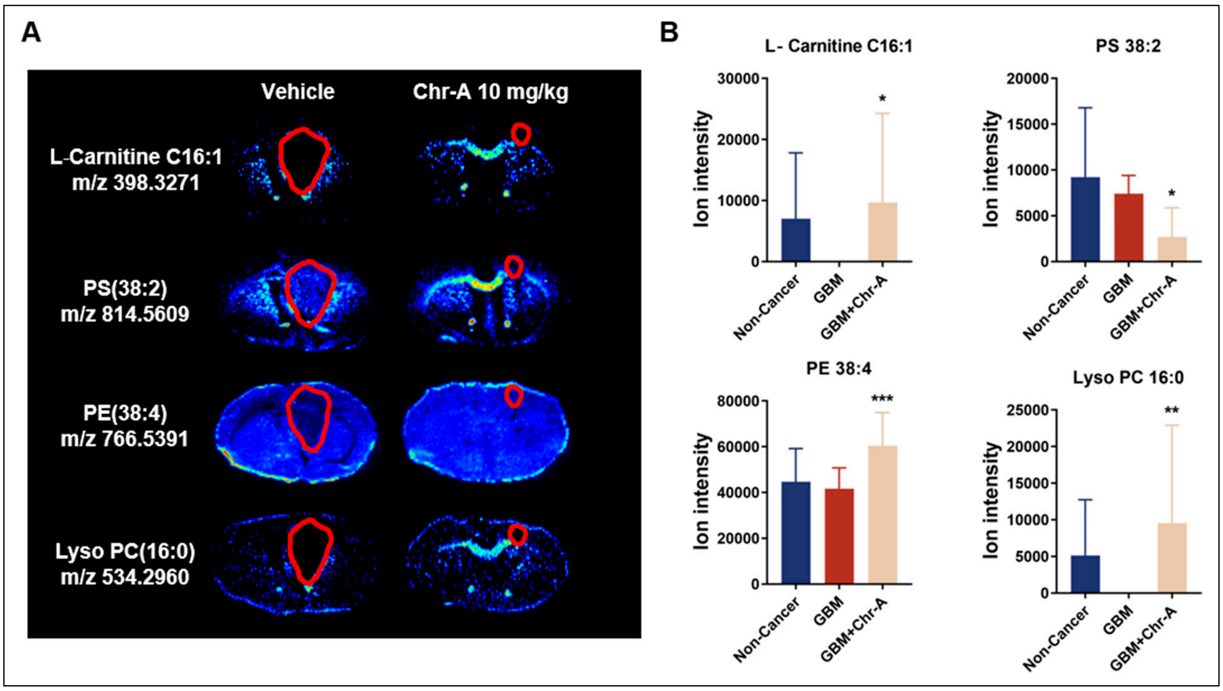

Figure S1. Region-specific MS images of L-Carnitine(C16:1), PS (38:2), PE (38:4) and lyso PC (16:0) in the glioblastoma intracranial model. (**A**) Region-specific MS images of L-Carnitine(C16:1), PS (38:2), PE (38:4) and lyso PC (16:0). (**B**) The ion intensity of L-Carnitine(C16:1), PS (38:2), PE (38:4) and lyso PC (16:0) in non-cancer tissue and GBM tissue with or without Chr-A. The red line areas represent distinct tumor tissue. All the data are presented as means ± SD from three independent experiments. ***p < 0.001, **p < 0.01, *p < 0.05 vs. GBM tissue.

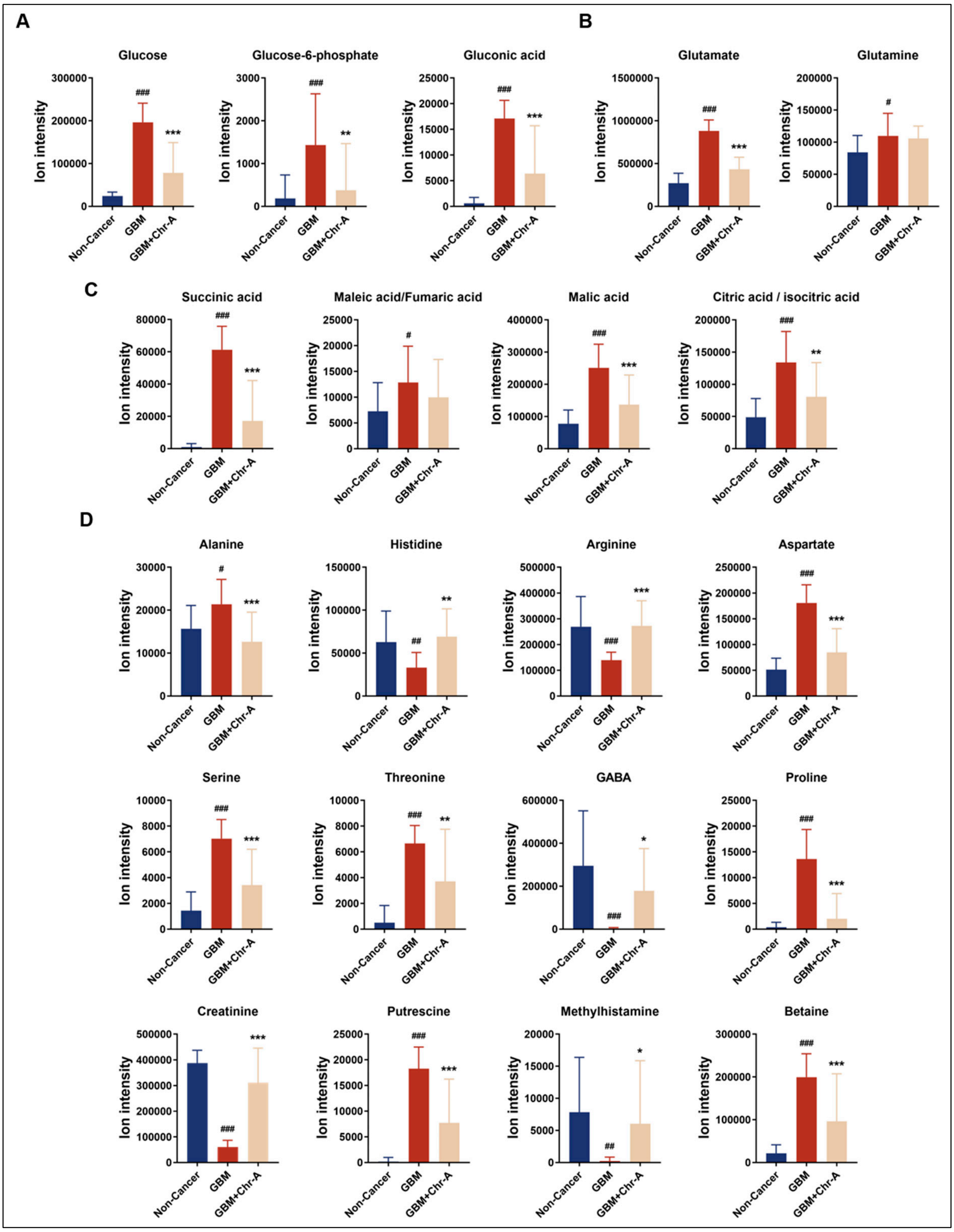

Figure S2. The ion intensity of metabolites in various metabolism pathway determined by AFADSI-MSI. The ion intensity of metabolites of glucose metabolism (A), metabolites of glutamine metabolism (B), metabolites of TCA cycle (C) and other amino acids (D) in non-cancer tissue and GBM tissue with or without Chr-A. All the data are presented as means ± SD from three

independent experiments. ###p < 0.001, ##p < 0.01, #p < 0.05 vs. non-cancer tissue, ***p < 0.001, **p < 0.01, *p < 0.05 vs. GBM tissue.

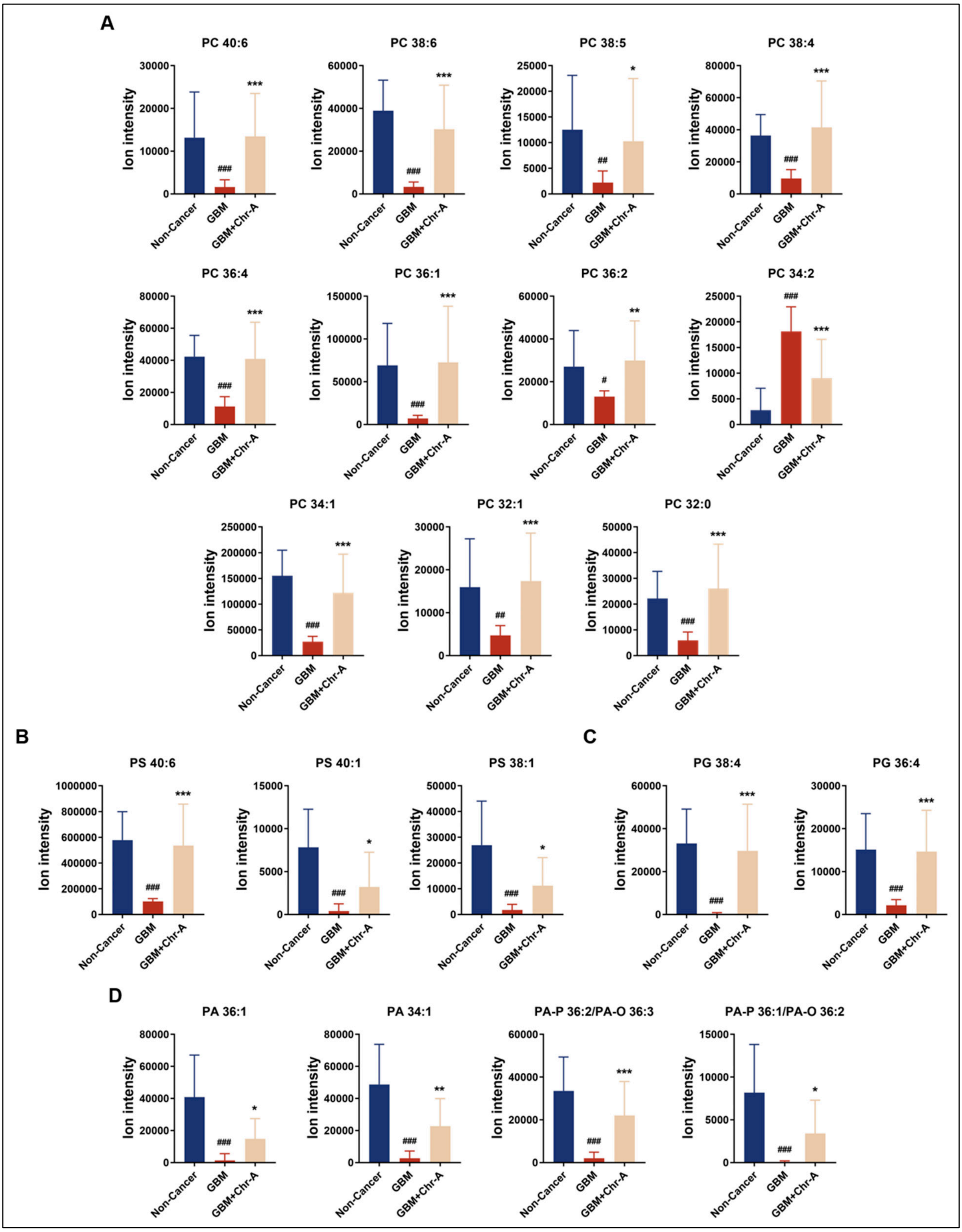

Figure S3. The ion intensity of PCs, PSs, PGs and PAs determined by AFADSI-MSI. The ion intensity of PCs (A), PSs (B), PGs (C) and PAs (D) in non-cancer tissue and GBM tissue with or without Chr-A. All the data are presented as means ± SD from three independent experiments.

###p < 0.001, ##p < 0.01, #p < 0.05 vs. non-cancer tissue, ***p < 0.001, **p < 0.01, *p < 0.05 vs. GBM tissue.

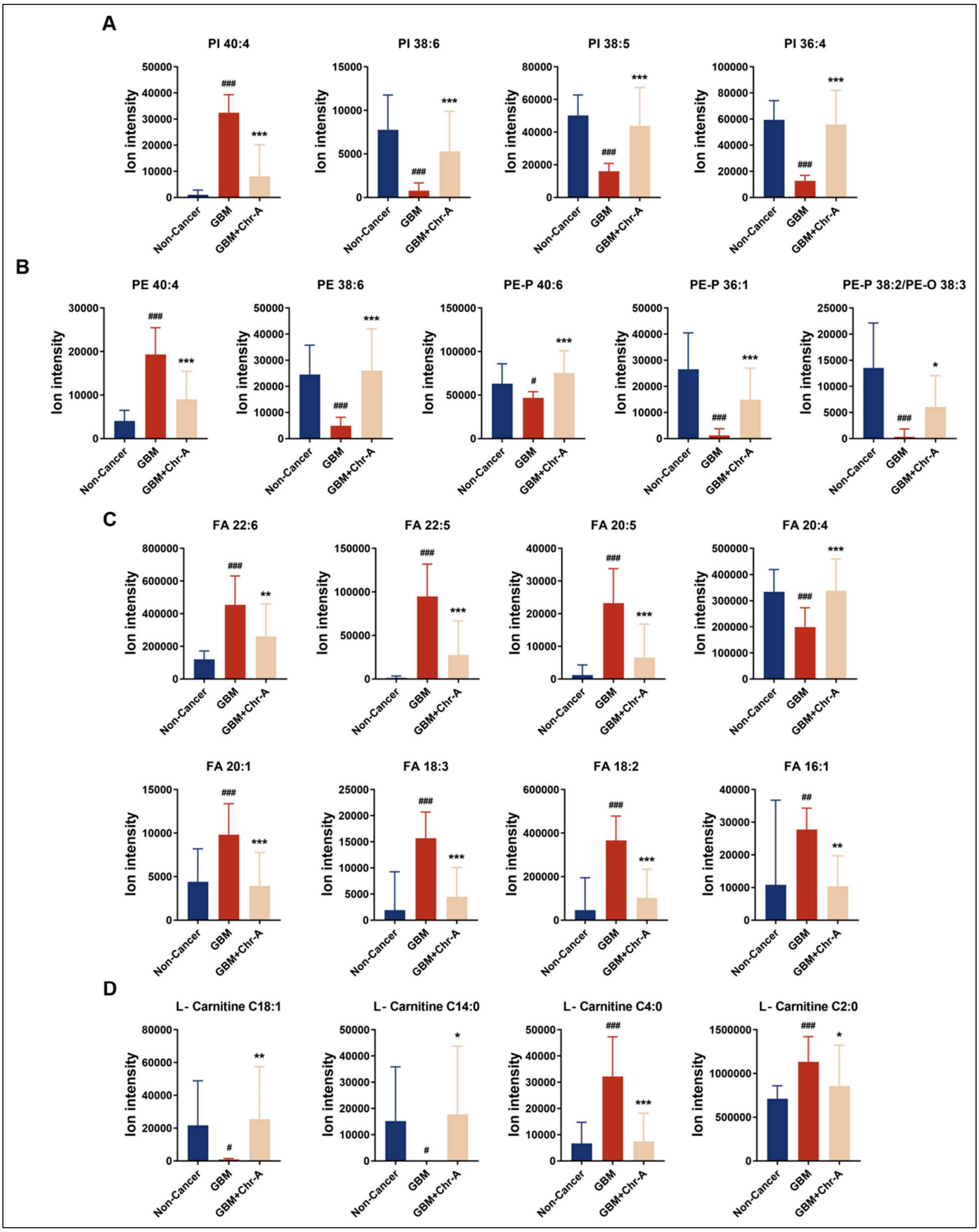

Figure S4. The ion intensity of PIs, PEs, FAs and L-Carnitines determined by AFADSI-MSI. The ion intensity of PIs (A), PEs (B), FAs (C) and L-Carnitines (D) in non-cancer tissue and GBM

tissue with or without Chr-A. All the data are presented as means ± SD from three independent experiments. ###$p < 0.001$, ##$p < 0.01$, #$p < 0.05$ vs. non-cancer tissue, ***$p < 0.001$, **$p < 0.01$, *$p < 0.05$ vs. GBM tissue.

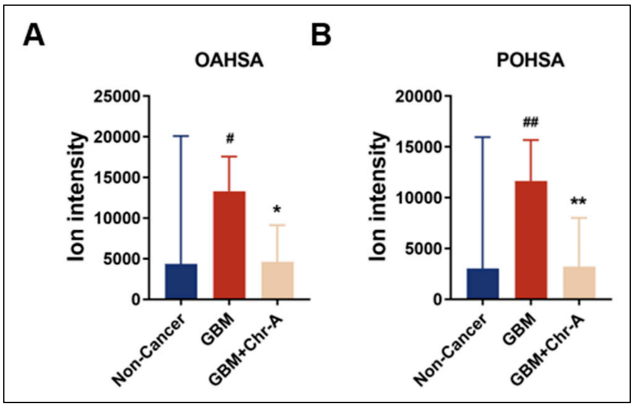

Figure S5. The ion intensity of OAHSA and POHSA determined by AFADSI-MSI. The ion intensity of OAHSA (A) and POHSA (B) in non-cancer tissue and GBM tissue with or without Chr-A.

Table S1. Antibody information used in the experiment

Antibody name	Product ID	Production company	Dilution ratio
GLS	56750	Cell Signaling Technology	1:1000
HK2	2867	Cell Signaling Technology	1:1000
G6PD	ab993	Abcam	1:1000
PKM2	4053	Cell Signaling Technology	1:1000
SOD-2	13141	Cell Signaling Technology	1:1000
GAPDH	2118	Cell Signaling Technology	1:1000
SOD-1	10269-1-AP	Proteintech	1:1000
Nrf-2	16396-1-AP	Proteintech	1:1000
HO-1	10701-1-AP	Proteintech	1:1000
NQO-1	11451-1-AP	Proteintech	1:1000
Catalase	sc-271803	Santa Cruz Biotechnology	1:500
GDH1	abs118006	Absin	1:1000

Table S2. Other agents' information used in the experiment

Agent name	Product ID	Production company
Temozolomide	HY-17364	MedChemExpress
Dulbecco's modified Eagle's medium (DMEM)	PM150210	Procell
Fetal bovine serum (FBS)	164210	Procell
Chrysomycin A	kindly provided by Prof. Hua-Wei Zhang (Zhejiang University of Technology)	
